# Cognitive Radio MANET Waveform Design and Evaluation †

**DOI:** 10.3390/s21041052

**Published:** 2021-02-04

**Authors:** Anna Kaszuba-Chęcińska, Radosław Chęciński, Piotr Gajewski, Jerzy Łopatka

**Affiliations:** Faculty of Electronics, Institute of Communications Systems, Military University of Technology, Gen. Sylwester Kaliski Str. No. 2, 00-908 Warsaw, Poland; radoslaw.checinski@wat.edu.pl (R.C.); piotr.gajewski@wat.edu.pl (P.G.); jerzy.lopatka@wat.edu.pl (J.Ł.)

**Keywords:** dynamic spectrum management, spectrum sensing, cognitive radio, waveform, ad hoc network testbed

## Abstract

The problem of waveform construction for mobile ad hoc networks with cognitive radio (MANET-CR) is discussed. This is the main limitation to widely use this very attractive technique, which does not need the deployment of expensive communication infrastructure. Two main questions correspond to MANET-CR effectiveness: spectrum sensing and spectrum sharing. The paper presents the structure of CR nodes that enables Opportunistic Spectrum Sharing. Procedures for advanced Dynamic Spectrum Management together with the concept of policy-based radio and a sensing method are presented. In the proposed system, the basic policy is to avoid interference generated by other users or jammers. The experiments were performed in a real environment, using the elaborated testbed. The results show that the use of sensing and cognitive management mechanisms enable more efficient use of the spectrum while maintaining reasonable overhead values related to the management procedures.

## 1. Introduction

Mobile Ad Hoc Networks (MANET) are gaining growing interest because they enable communication between radio nodes without infrastructure. This technology is used in the case of systems consisting of a variety of network elements such as wireless nodes including vehicular, personal, and temporary fixed (but nomadic) platforms, as well as wireless sensors. All these nodes create a robust and reliable network of networks. Such an approach is widely used in military and governmental systems and may be used in future 5G systems working in device-to-device mode.

The main goal of this paper is to present the developed MANET cognitive waveform with spectral agility, enabling mitigation of interferences, improvement of network reliability, and throughput. The waveform is implemented in a real-time demonstrator to enable its validation in a real spectral environment.

Spectrum scarcity is a basic limitation for the extensive use of such networks that are the area of interest for both military and civilian applications. Limited spectral resources enforce the use of complex dynamic spectrum management procedures in which Cognitive Radio (CR) is the most promising solution.

Cognitive radio is defined as an intelligent radio, which is based on Software Defined Radio (SDR) technology that is aware of its electromagnetic environment and can react to input stimuli to perform a reliable communication system and maximize spectrum utilization [1]. The main issue in commercial radio systems is increasing user demand for throughput related to a scarcity of available spectrum resources. For this reason, dynamic spectrum access mechanisms [2,3,4] are strongly developed. Military communication as tactical radio networks, in addition to these issues, must deal with intentional jamming and interferences. In this case, different types of jammers are used. These are classified in [5]. The most widely known types of jammers are spot and barrage jammers. The first one attacks a specific frequency and the second one jams a range of frequencies.

The most popular cognitive technique to mitigate jamming for small mobile platforms with a single antenna is the change in the transmission channel based on spectrum sensing results.

Conventional transmission techniques, e.g., Frequency Hopping (FH) [6] or Direct Sequence Spread Spectrum (DSSS) [7,8], are used to counteract jamming, but without spectrum monitoring, any devices using these techniques are not aware of available alternative resources.

Recently, Internet of Things (IoT) devices operating in the ISM 2.4 GHz and 900 MHz bands are gaining popularity, which may pose a threat to the free spectrum access. Additionally, in the 2.4 GHz band, it is necessary to share spectral resources with numerous devices transmitting under the 802.11 (Wi-Fi) standard. In most cases, IoT devices transmit data using the 802.15.4 standard, which is dedicated to use Carrier Sense Multiple Access with Collision Avoidance (CSMA/CA), which prevents collisions with other devices. However, it does not solve the problem of hidden nodes and can introduce delays in the case of too many interferences. In 2012, an additional Time Slotted Channel Hopping (TSCH) [9] mode was introduced as an amendment (802.15.4e) to the Medium Access Control (MAC) of IEEE 802.15.4. In this mode, access to the medium is organized using Time Division Multiple Access (TDMA) and the Frequency-Hopping Spread Spectrum (FHSS). This allows us to increase the efficiency of the transmitted data and reduce the impact of fast-fading effects on the quality of transmission. In the TSCH, nodes transmit data in each time slot, and the center frequency changes them. To avoid interferences from other systems, this mode provides a list of forbidden channels (blacklist) for which the Quality of Service (QOS) may be unacceptable. Unfortunately, the standard does not define how to build this list in specific cases and currently it is an open issue [10].

On the other hand, in the case of a cluster-based MANET with FH, it requires orthogonal codes and sequence modification to assure no interferences take place between clusters. This increases the complexity of the system and may also lead to collisions. Other issues related to MANET are continuous interferences that can jam the traffic that controls in the network. Nodes do not recognize if it is a temporary interference and should stay at the jammed channel for some time, or whether it is intentional interference, and they should start the procedure of searching for a new free channel. As a result, new synchronization in the network must be performed.

To ensure effective spectrum access, without collision with other radio systems, it is important to have precise and instantaneous knowledge about spectrum usage. Since the urban environment is very diverse, and, in some cases, signals can be detected in one place only to disappear a few meters away, cooperative sensing [11] seems to be a good solution for overcoming these issues [12].

There are two main approaches in cooperative sensing: wideband and narrowband sensing. Wideband sensing allows us to analyze signals in larger numbers of adjacent channels at one time, while narrowband sensing allows detection of narrowband signals [13]. The commonly used energy detector is easy to implement and introduces few delays, but it does not handle signals with low signal-to-noise ratios well. Cooperative sensing increases the probability of detection in this case.

Cooperative sensing can be performed with soft or hard decision making. In the case of the first one, signal samples are sent to main nodes from every cooperative node. This improves the decision algorithm but decreases the throughput in the network for user services. A compressive sensing approach [14] can be used to overcome these limits. However, these methods can be computationally complex and require high sampling rates and high-resolution analog-to-digital converters. In the hard decision method, only sensing results (free or busy channel) are sent to main nodes. A disadvantage of this method is the lack of knowledge about the percentage channel occupancy.

This paper addresses the question of what communications and networking technology can be used to fully realize data exchange by handheld radios in a complex radio-environment in the battlefield. It is an extension of work described in [15]. The concept of the waveform, which can prevent intentional jamming and interferences from other systems, has been demonstrated. This solution is based on a cognitive radio network with cooperative sensing, where all nodes in the network detect interferences in backup channels and send results to the main node in a cluster. The main node, based on these data, sets a list of the available backup channels for the cluster. The demonstrated implementation of this waveform consists of a cognitive plane and a Basic Waveform (BW) plane. BW is based on techniques currently used for MANET solutions corresponding to the Orthogonal Frequency Division Multiplexing (OFDM) modulation [16] in the physical layer and 802.15.4 [17] standard for the MAC layer.

Our main contributions are:

- adaptation of 802.15.4 MAC frames for cognitive spectrum management,
- proposition of multi-channel sensing for devices with one radio frequency interface,
- best channel selection method for optimal spectrum access,
- creation of a testbed for MANET waveform development.

Section 2 presents the global characterization of the CR waveform construction for MANET. Proposed sensing methods are presented in Section 3. Section 4 introduces the architecture of the implemented cognitive waveform on the SDR platform. In Section 5, the authors describe the elaborated radio network testbed. Results are shown in Section 6, while Section 7 presents the conclusion and future work.

## 2. CR Waveform Construction

### 2.1. The Key Challenge of CR MANET Implementation

Two main questions correspond to CR MANET implementation. The first one is how to recognize spectrum occupancy and to decide if the frequency channel is free or not. The sensing process should be accurate in terms of the minimal signal level detection and occupancy decision probability and should rapidly monitor the possible wide spectrum band.

The second question concerns the network organization and used procedures. Here, the following issues are the main key challenge for CR MANET implementation:

- Dynamically discover, authenticate, and connect
- Autoconfiguration capabilities with self-organizing mechanisms
- Routing exchange compression
- MAC protocols
- Network recovery
- Security and vulnerability

### 2.2. State of the Art Analysis of CR Waveform Implementation

**Figure 1:**
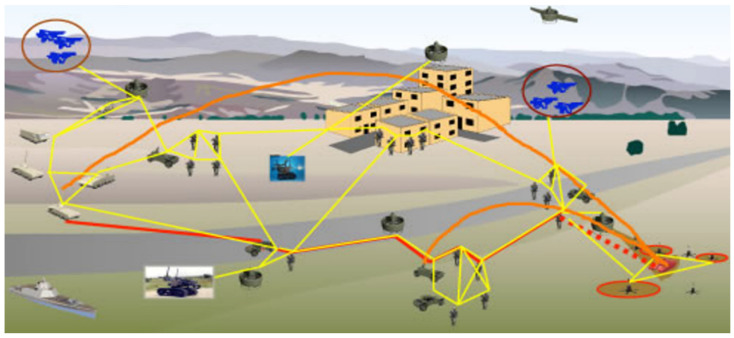
Military Tactical Networking.

Below, a short overview of recent work achieved in mentioned challenges is presented. It can be stated that references discuss mostly some questions concerning CR MANET that could be used for communication supporting highly dynamic military operations, where the heterogenous networking system is used (Figure 1), including Wireless Sensors Networks (WSN).

**Figure 2:**
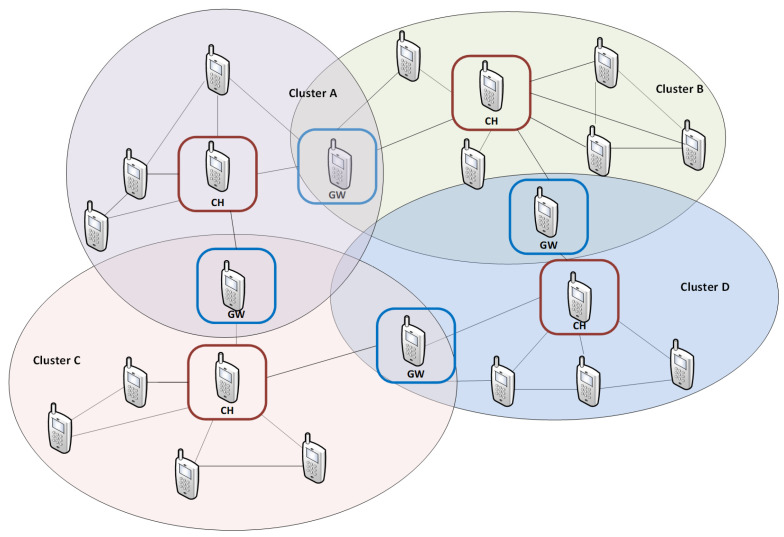
Cluster network example.

Military MANET must meet four general requirements [18]: Strong Connectivity, Very High Bandwidth, Effective Security, and Survivability. Such a heterogenous network is created as a set of clusters with homogeneous nodes. The hierarchical management process is used in cluster topology (Figure 2).

Such a network includes three types of nodes: Cluster Head (CH), which is responsible for resource management within the cluster, Gateway (GW), which provides communication between neighboring clusters, and Regular Node (RN). Each of them uses one spectrum resource, other than the neighboring one. This phenomenon corresponds to the cluster coloring. Each node can be used as a gateway in multihop transmission between clusters.

In cluster creation, the CH should be selected adequately. In [19], the authors present the concept of the Weighted Clusterhead Node Election algorithm (WCNE) in a MANET. In the algorithm, a CH set is chosen that minimizes the energy consumed for transmission, while maximizing the network’s lifetime and the probability of delivering information to the sink. The specific weight components are related to the mobility, battery level, and received signal-to-noise power ratio for each node. Furthermore, energy limitation is also discussed in [18]. Here, the Low Energy Adaptive Clustering Hierarchy (LEACH) protocol and Stable Election Protocol (SEP) are compared and the authors propose Advanced SEP to improve the lifetime of sensors in MANET. Energy efficiency is also the main objective discussed in [16] for OFDM systems, where lifetime and reliability are the main criteria for WSN due to the limitation of energy sources. These questions are reviewed widely in [20,21].

Due to the network mobility, its structure is variable, and it should be adapted according to the results of neighbors’ discovery. For this purpose, HELLO messages are used, and they can be modified to enable adaptive network reconfiguration. An enhanced superframe structure for real time data transmission is proposed in [22] for networks based on the IEEE 802.15.4 standard. This standard is suggested in [17] for Dynamic Spectrum Access (DSA) in SDR-based networks. Issues concerning modelling and optimization of the IEEE 802.15.4 protocol for reliable and timely communications are described in [23], and this standard network performance and trade-offs are discussed in [24]. In [25], the reliable communication model based on this standard is assumed for WSNs in a Smart Grid. Some adaptations of the IEEE 802.15.4 standard are used in our solution and they are described in Section 4.

The variable structure of the network is also challenging for routing. Various routing protocols were recently studied and proposed in term of CR MANET. The paper [26] presents a performance evaluation of IETF MANET routing protocols (DYMO, OLSR, and AODV) against a mobility model of nodes using the NS2 discrete network simulation tool. A wide comparison of routing protocols proposed for use in WSN is discussed in [27]. For short distance CR networks, the Enhancement of Opportunistic Ad-hoc On Demand Distance Vector (EOAODV) is proposed in [28]. Here, the next hop selection is based on the signal level of the neighboring nodes, shortest distance, and the lowest Expected Transmission Count (ETC).

The context-aware routing mechanisms are also studied; such a method is proposed by [29] for a standalone communication system using contextual information as delivery probability and link quality. The link quality is a metric often proposed as a criterion used in MAC protocols which has been discussed in [30]. This paper presents a summarized study of various MANET algorithms and protocols to find the best available path and travel data along the network to the destination.

Efficient Dynamic Spectrum Management balances the energy consumption problem, eliminates conflicts between the nodes, reduces the channel interference, and divides the traffic over different channels and time slots. In [31], a new channel management algorithm for Cognitive Radio Sensor Networks is proposed, which increases the energy efficiency using a hidden Markov model (HMM). The proposed algorithm adaptively selects its operation mode among channel sensing, channel switching, and data transmission/reception, according to the channel-sensing outcome.

A review of the state-of-the-art MAC protocols focused on CRAHN is presented in [32] in the context of free spectrum recognition, available resource scheduling, and coordination of heterogeneous systems and users.

Another important issue in CR MANET implementation concerns security aspects. This is a multilevel problem connected with the vulnerability of any kind of actions (including attacks) against communication, information, network, nodes, and users. One of the problems is to build a system of trust for nodes (users) that cooperate with each other, having an important influence on the cognitive system behavior. The Distributed Denial-of-Service (DDOS) is an example of persistent attack that affects the network behavior. In [33], a Machine Learning technique is proposed to detect DDOS attack. Another issue concerning a comparison of symmetrical key algorithms for IoT devices is discussed in [34].

Military CR MANET is designed to handle traffic with very different characteristics, from short, periodically sent messages with Blue Force Tracking (BFT) containing position data, up to video transmission with high randomness of duration, that requires high throughputs between nodes. Two problems are related to traffic. The first one is devoted to traffic control, and the second one relates to traffic models used in evaluation. The latter have a significant impact on the results of CR network performance. In [35], the traffic analysis using MANET in WSN is provided in a context of anonymous routing protocols relying on hop-by-hop encryption or redundant traffic. A statistical traffic pattern discovery system is proposed. Another proposal for traffic control is presented in [36]. This Min-Max Scheduling Load Balancing (M2SLB) improves the network throughput, packet delivery ratio, and minimizes delay and packet loss.

### 2.3. Software Process for CR Waveform Design

The development of CR consists of two phases: specification and validation. In the first step, the requirements for the CR network are defined and their capabilities are specified. It also concerns the choice of AI procedure implementation with algorithmic Machine Learning and statistical decision making. At the beginning, we assumed implementing clustered network as better matched to hierarchical command organization [37].

In the second phase, coded blocks of the waveform are validated during unit tests and finally, global solutions are assessed in integrated tests. In general, each node in a MANET generates multiple streams of packets that are directed to other nodes. These streams relate to user and traffic signaling with different quality requirements depending on their type. Network performance can be measured at all layers of the communication stack. However, usefulness of specific metrics, cost related to necessary computation effort, and consumption of resources for results transmission are different.

A set of specific metrics was used in [35] to assess a MANET WSN performance. This evaluation contains characteristics of packet drop, throughput, and packet delivery ratio in time showing an impact of disturbance on the network. Other parameters were used in [38] for the evaluation of various network traffic impacts on characteristics of CR networks based on the IEEE 802.22 standard.

In our studies, two types of metrics are analyzed: metrics for the ongoing evaluation of transmission parameters, showing their changes over time, called time-dependent metrics. In addition, average metrics are used to evaluate the link parameters, called per stream metrics. The most important metrics selected from this set to assess our solution are described below. The calculation formulas were taken from [39] where the wide set of metrics proposed for the evaluation of MANET CR is discussed.

#### 2.3.1. Time-Dependent Metrics

For instantaneous evaluation of transmission performance, three metrics were chosen, namely: temporary Received Signal Strength Indicator (RSSI), instantaneous value of Packet Error Rate (PER), and instantaneous value of throughput–R. The calculation formulas are as below:

- temporary RSSI (RSSI(t)) of the *r*-th packet stream sent by the *i*-th node and registered in the *j*-th node [dBm]:

(1) $$RSSI_{ij}^{r}\left(t\right)=10\log_{10}\left(\frac{Prec_{ij}^{r}\left(t\right)\left[\mathrm{mW}\right]}{1\left[\mathrm{mW}\right]}\right)$$

where: $Prec_{ij}^{r}\left(t\right)$—received signal power, measured in the *j*-th node at a moment t of packet reception; for packets belongs to the *r*-th stream sent by the *i*-th node [mW].

- instantaneous value of PER (PER(t)) of the *r*-th stream between the *i*-th and *j*-th node:

(2) $$PER_{ij}^{r}\left(t\right)=\frac{L_{ij}^{r}\left(t\right)}{D_{ij}^{r}\left(t\right)}\;$$

where: $L_{ij}^{r}\left(t\right)$—number of lost frames in Δt intervals registered at the end of each interval during *r*-th stream transmission between the *i*-th and *j*-th node, $D_{ij}^{r}\left(t\right)$—number of packets generated by the *i*-th node and sent to the *j*-th node in Δt.

- instantaneous value of stream throughput (R(t)) of the *r*-th stream [bits/s]

(3) $$R_{ij}^{r}\left(t\right)=\frac{Nb_{ij}^{r}\left(t\right)}{\Delta t}$$

where: $Nb_{ij}^{r}\left(t\right)$—number of bits correctly sent in Δt intervals [s] in the *r*-th stream between the *i*-th and *j*-th node registered at the end of each interval [bits].

#### 2.3.2. Per Stream Metrics

To link the evaluation, the mean values of PER, throughput R, and traffic percentage for neighbor discovery *Rm_hello*, and for sensing *Rm_sensing* were used. The formulas used for calculation are:

- mean PER (PERm) of the *r*-th stream between the *i*-th and *j*-th node:

(4) $$PERm_{ij}^{r}=\frac{L_{ij}^{r}}{D_{ij}^{r}}\;$$

where: $L_{ij}^{r}$—number of lost packets in the *r*-th stream between the *i*-th and *j*-th node, $D_{ij}^{r}$—number of packets generated by the *i*-th node and sent to the *j*-th node.

- mean stream throughput (Rm) of the *r*-th stream between the *i*-th and *j*-th node [bits/s]:

(5) $$Rm_{ij}^{r}=\frac{Nb_{ij}^{r}}{\Delta t}\;$$

where: $Nb_{ij}^{r}$—number of bits correctly sent between the *i*-th and *j*-th node in the *r*-th stream [bits], Δt—observation time interval [s].

- neighbor discovery traffic percentage (Rm_hello) in the *r*-th stream between the *i*-th and *j*-th node [%]:

(6) $$Rm\_hello_{ij}^{r}=\frac{\left(\frac{R\_hello_{ij}^{r}}{\Delta t}\right)}{Rtm_{ij}^{r}}\cdot 100\%\;$$

where: $R\_hello_{ij}^{r}$—number of bits of neighbor discovery messages correctly sent between the *i*-th and *j*-th node [bits], Δt—observation time interval [s], $Rtm_{ij}^{r}$—mean throughput of the *r*-th stream [bits/s].

- sensing traffic percentage (Rm_sensing) in the *r*-th stream between the *i*-th and *j*-th node [%]:

(7) $$Rm\_sensing_{ij}^{r}=\frac{\left(\frac{R\_sensing_{ij}^{r}}{\Delta t}\right)}{Rtm_{ij}^{r}}\cdot 100\%\;$$

where: $R\_sensing_{ij}^{r}$—number of bits of sensing data correctly sent between the *i*-th and *j*-th node [bits], Δt—observation time interval [s],$\;Rtm_{ij}^{r}$—mean throughput of the *r*-th stream [bits/s].

## 3. Multichannel Sensing

Spectrum sensing is an immanent process of Cognitive Radio that enables us to determine which part of the spectrum is unused and which is occupied by other users: primary users and secondary users, or which part of the spectrum is jammed. Classical theory shows that a matched filter is optimal to detect known signals. If the signal is unknown, the Energy Detector (ED) is most often used because of its low computational complexity and implementation simplicity [40].

In the presented solution, multichannel sensing is proposed. Here, the monitored frequency range is divided into a set of channels using a filter bank. Each output of this bank is connected with ED. A Weighted Overlap-Add (WOLA) filter bank is proposed in [41]. The WOLA filter algorithm realizes calculations for L samples of the signal located in the L-length buffer; next, the results of sample multiplication with a filter impulse response are divided into L/K blocks of K samples; and K-point Fourier transform is computed using the sum of ordered samples from each data block. ED computes the signal energy in the time domain using the formula below:

(8) $$E=\sum_{i=1}^{N}{\left|y\left(i\right)\right|}^{2}\;$$

Here, the output of ED can be the minimum energy of a sample from the set specified by the number of signal time slots, the maximum energy of the sample from the set specified by the number of signal time slots, the average energy for a single sample, and the energy for a certain number of time slots.

**Figure 3:**
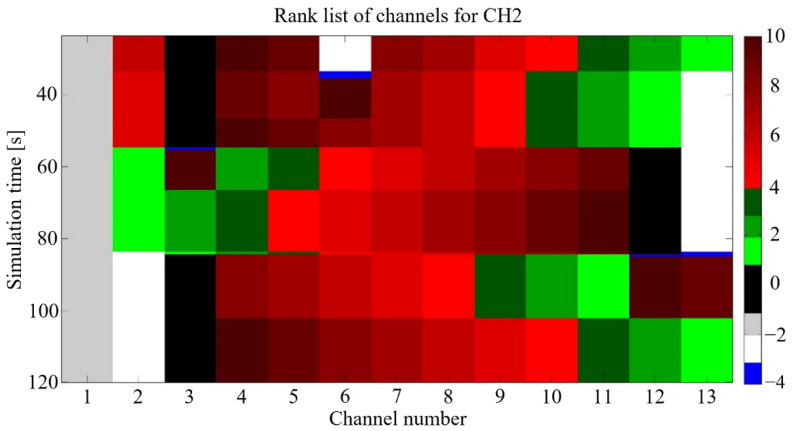
Simulation results with ranking list of channels—an example.

Knowledge of the noise power is required to properly set the decision threshold, which is the main disadvantage of the ED, because the channel conditions vary and change in each node location, so the sensing decision can also vary inside a cluster. To prevent this, cooperative sensing inside clusters is proposed by the authors [42]. Here, the individual measures from nodes are sent to the CH that performs algorithm data fusion, makes the channel ranking list, and selects the best channel as a backup. CH decisions are then distributed within the cluster. An example of a ranking list is shown in Figure 3.

A proposed algorithm for ranking list creation is described in [42]. It is based on fitness parameter values, which are calculated for all channels according to the following formula:

(9) $$F=w_{1}\cdot O+w_{2}\cdot D+w_{3}\cdot Q$$

where: F—is channel fitness, O—is occupancy measure, D—is distance measure, Q—is link quality measure (learning), $w_{1}$—is occupancy measure weight, $w_{2}$—is distance measure weight, $w_{3}$—is link quality measure weight (learning).

## 4. CR Waveform Composition

### 4.1. CR Waveform Architecture

The implemented waveform is intended to work in a mobile network topology, organized in clusters, where each node can work as a CH or a RN. Each node has implemented a basic waveform (responsible for data transmission in the network) and cognitive elements: Sensing Module (SM) and Cognitive Manager (CM). SM is responsible for outband spectrum monitoring of predefined channels and preparing results for the CM, which is based on sensing results from all nodes in a cluster, and the PER metric for all node relations selects the best data channel for transmission.

**Figure 4:**
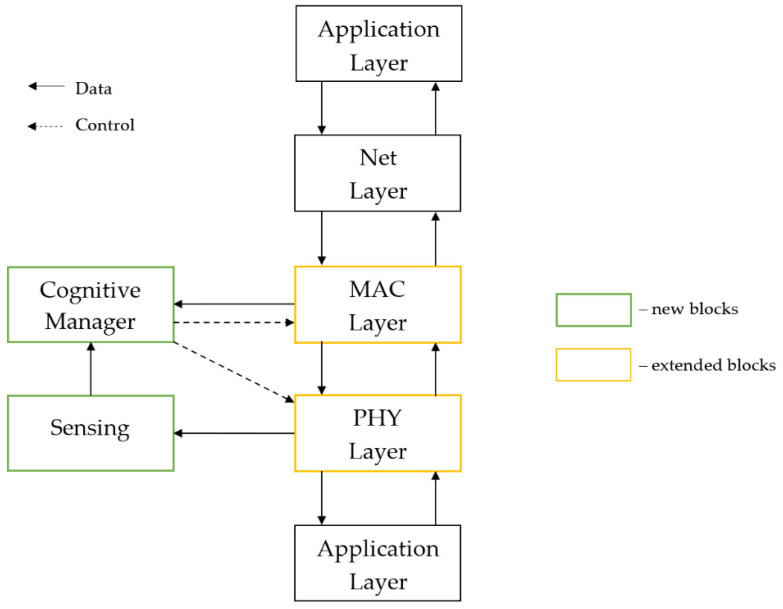
The architecture of the cognitive waveform.

The architecture of the cognitive waveform is presented in Figure 4. It is adapted for implementation on the Software Defined Radio platform. The basic waveform is implemented with four layers of the ISO Open Systems Interconnection Reference Model: the physical layer (OFDM modulation/demodulation, encode/decode, etc.), MAC layer (managing radio access, generating TDMA frames), network layer (route packets from one node to another during non-line of sight condition), and application layer (data streaming in continuous or cyclic mode). Each layer consists of a transmitter and receiver path. Additionally, the first two layers include implemented managers for communicating with the cognitive manager. The physical layer has two additional interfaces. The first one is connected to a sensing module to enable the transfer of IQ samples from the device. The second one handles communication with the Universal Software Radio Peripheral (USRP) device via a USRP Hardware Driver (UHD).

Therefore, the waveform works with a 2 MHz sampling rate and the transmitted OFDM signal has a bandwidth of 1 MHz. Each subcarrier is modulated using a QPSK scheme. The MAC layer is based on TDMA, which is often used in MANETs. The authors assumed that the waveform should work with one RF tuner; therefore, in the TDMA frame, 8 slots were defined for user data transmission and 2 slots for sensing. In the described solution, TDMA frame parameters depend strictly on USRP frequency tuning time limitation.

Waveform parameters (Table 1) were selected to reflect the parameters of real waveforms.

In the presented implementation, the authors focused only on dynamic spectrum allocation; therefore, mechanisms such as buffering, queuing, packet segmentation and reassembly, packet retransmission, etc. are not implemented. Additionally, the network layer includes a simple router to route user data to the one-hop neighbor.

The following sections explain the types of frames used in the presented waveform.

#### 4.1.1. Physical Layer

The Physical Layer (PHY) is implemented using the liquid-dsp library. The PHY frame consists of preambles for time and frequency synchronization, headers including the PHY frame number, and payload. Frames coming from the MAC layer are encoded with binary Golay code, and a 32-bit Cyclic Redundancy Check (CRC) is also performed.

#### 4.1.2. MAC Layer

MAC frames implemented in the presented waveform are based on the 802.15.4 standard [43]. For cognitive dynamic allocation purposes, the authors modified the beacon frame and added new frames. Their structures are described below.

**Figure 5:**
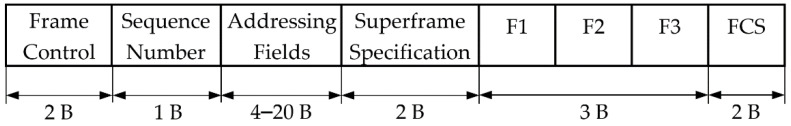
Beacon frame format.

**Figure 6:**
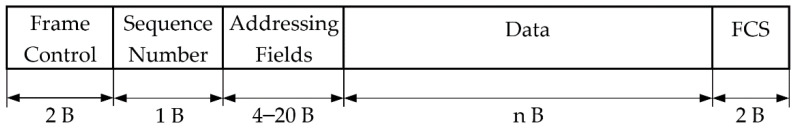
Data frame format.

**Figure 7:**
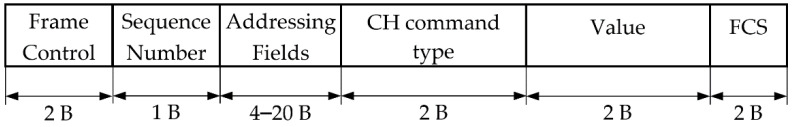
CH command frame format.

**Figure 8:**
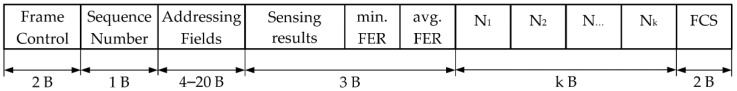
Hello frame format.

**Figure 9:**
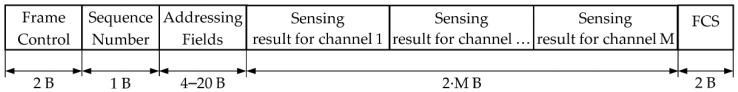
Sensing frame format.

**Figure 10:**
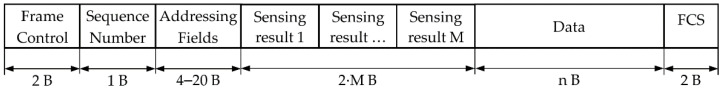
Data-sensing frame format.

1. The beacon frame (Figure 5) is sent only by the Cluster Head to perform network synchronization and inform other nodes in the cluster about backup channels, which can be potentially used by CH after the completion of the data channel switching procedure. In relation to the standard, there are no Guaranteed Time Slot fields and Pending address fields. F1, F2, and F3 fields contain indexes of three backup channels (each node has the same channel list).

2. Data frame (Figure 6) is defined by the 802.15.4 standard.

3. The CH Command (Figure 7) frame is not defined in the aforementioned standard. It is used for sending control commands from CH to regular nodes. It contains fields for command type and value. The main goal of this frame is to provide channel switching information to a node (in this situation, the value represents an index of a new data channel).

4. The hello frame, not defined in the 802.15.4 standard, is responsible for neighbor discovery. Each node sends this frame in defined intervals (during the tests this interval was set to three seconds). Hello frames contain information about (Figure 8):
  - sensing results (1 byte)—every bit contains information about channel occupancy,
  - minimum frame error rate FER (1 byte)—the minimum value of FER calculated for all node relations (FER is quantized. Level of quantization equals 256),
  - average FER (1 byte)—average value of FER calculated for all node relations, N1, N2, N_k_—one-hop neighbors MAC addresses, where N1 is an address of the first one-hop neighbor and N_k_ is the address of the k-th one-hop neighbor.

5. The sensing frame (Figure 9), not defined in the 802.15.4 standard, is used for transmitting sensing results from the regular node to the CH. There are defined Sensing Results Fields (SRF), of which the size is 2 bytes. The number of SRFs depends on the number of available data channels. The first field represents information about the first channel from the list (each node has the same channel list). Each field contains information about:
  - channel occupancy—1 bit (0 for free channel, 1 for detected signal),
  - data channel—1 bit—informed if this channel was used by the node for data transmitting or sensing (0—sensing, 1—data)—if this bit is set to 1, CH does not use this field for backup channel list calculations,
  - percentage occupancy of the channel (7 bits).

6. The data-sensing frame (see Figure 10), not defined in the 802.15.4 standard, is used for transmitting sensing results and user data from the regular node to the CH. The structure of this frame is similar to that of the sensing frame. Additionally, there is an added DATA field.

#### 4.1.3. Network Layer

The network layer is used only for routing data to a one-hop neighbor when the destination node is out of range. For this reason, a simple Net frame is defined, which contains:

- Destination address field,
- Source address field,
- Hop counter.

#### 4.1.4. Application Layer

To investigate whether the implemented waveform works correctly, the authors developed two types of services:

- BFT–used for transmitting short packets in a fixed interval,
- data services–transmitting data with maximum available throughput.

### 4.2. Cognitive Modules

The developed cognitive blocks are responsible for cooperative spectrum sensing, performed in dedicated sensing slots of the TDMA frame, and realization of the proposed proactive algorithm, based on the best alternative channel selection by the cluster head, and its dissemination between cluster members. The algorithm also contains a search procedure, enabling fast restoration of the cluster operation in the case of jamming.

The cognitive manager was implemented in all nodes in the network. It can be configured in two modes: RN or CH mode. In the RN mode, CM is responsible for collecting information from the sensing module and the basic waveform, processing them, and preparing results for sending to CH. It also reacts to received commands from CH (e.g., changing the data channel). CM in CH mode collects metrics from all nodes and chooses the best channel for transmission. Control frames between regular nodes and CH are sent via the same channel as user data; therefore, each node must monitor its own channel to be sure that it is not jammed. For this reason, energy detection is performed on each slot. Sensing and PER results are sent to CH. Based on these metrics and information from the MAC layer, CM can assign the slot to one of three states: no transmission, own transmission, the channel is jammed.

CM changes the data channel in the case of two events:

- when it detects that its own channel is being jammed,
- when the threshold of the average PER calculated for all relations in the network is exceeded.

The monitoring of outband channels was performed by the SM that uses the energy detection method, with the WOLA filter that splits one channel into a defined number of subchannels. Energy detection is performed for each of them. This method increases information about the degree of channel occupancy and allows us to detect narrowband signals.

The efficiency of the proposed method was experimentally verified, and results are included in the paper.

## 5. Testbed

**Figure 11:**
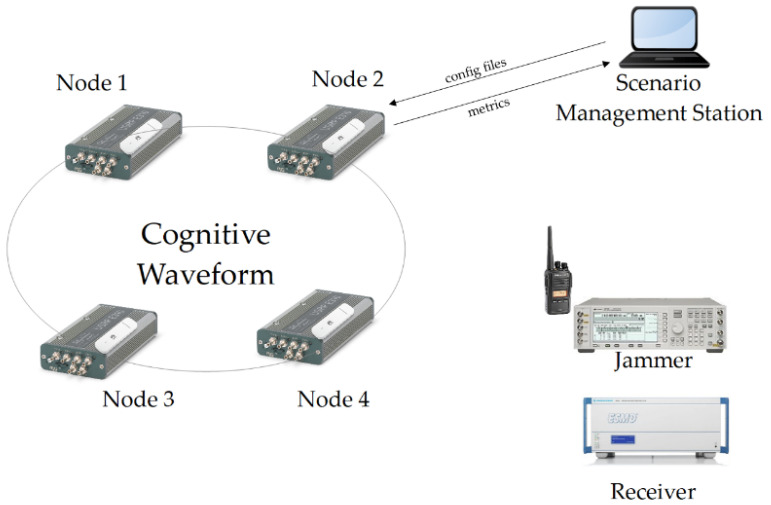
Testbed architecture.

The architecture of the testbed is presented in Figure 11. The testbed was prepared to emulate real radio environmental conditions for soldiers equipped with mobile radios, realizing specific scenarios such as patrol, base/convoy protection, etc. All tests were performed using a real-time demonstrator consisting of four Radio Nodes, a Scenario Management Station, Jammer Station, and Receiver.

**Figure 12:**
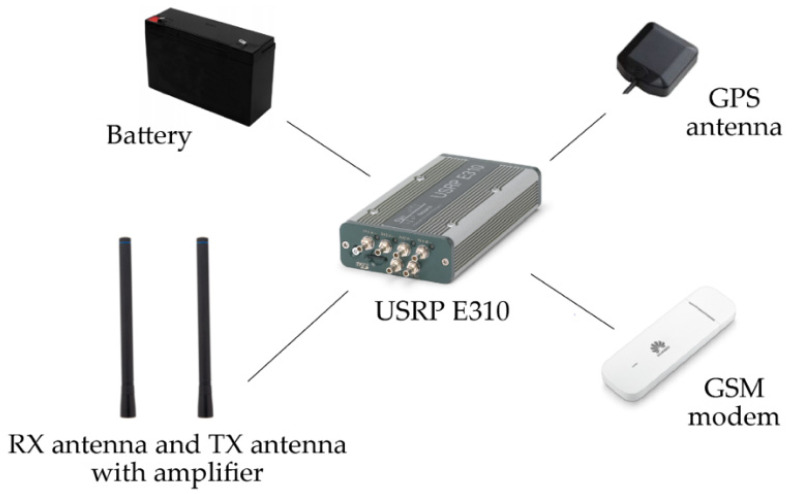
Radio node components.

Each radio node (see Figure 12) consists of a USRP E310 device. It is an embedded platform with GPP, GPS, and an RF tuner. Each node was equipped with a battery, TX amplifier, and GSM modem (used for receiving scenario configuration files and reporting node status). The implemented waveform was run on GPP.

The initial node configuration was performed by a Scenario Management Station, which allows the user to select the scenario and send configuration files with waveform parameters to all nodes (for instance, available channels). During the tests, the components were mounted on a vest, dressed by scenario participants, walking according to the established route. A signal generator and Personal Mobile Station were used for the generation of jamming signals. As a jamming signal, a typical 25 kHz FM signal was used. The testbed also consisted of a Rohde & Schwarz ESMD receiver for radio monitoring and spectrum recording.

**Figure 13:**
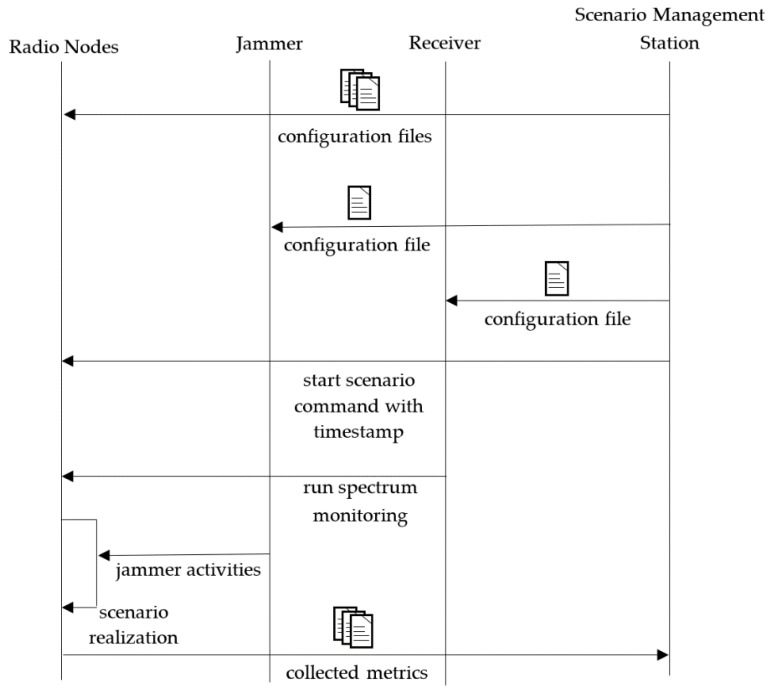
Sequence diagram of scenario realization.

The performed test was conducted in the following way (Figure 13):

1. A scenario with planned node mobility in an urban environment for four pedestrians was created.
2. Short messages (BFT) were exchanged between nodes.
3. The following parameters were collected over GSM during scenario realization:
  - GPS positions
  - Node activity
  - Number of neighbors
  - Channel number
4. After scenario realization, the following metrics were calculated:
  - Sensing results
  - Used radio channels
  - RSSI
  - Received packets

The setup is flexible and may be used for different use cases defined by the users.

## 6. Test Results

**Figure 14:**
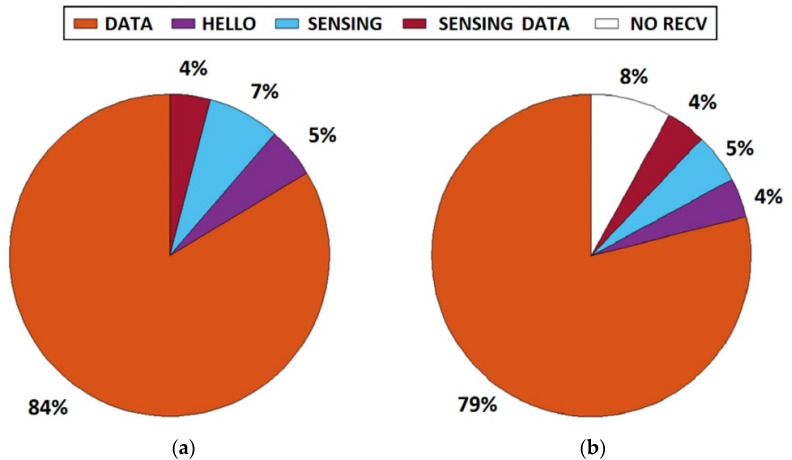
Packet type exchange between nodes 2 and 1: (**a**)—sent from node 2, (**b**)—received by node 1.

The percentage of packets type exchanged between nodes 2 and 1 is presented in Figure 14, according to the types of packets described in Section 2. During the test, data were sent continuously; 16% of all packets were dedicated to performing dynamic spectrum access.

**Figure 15:**
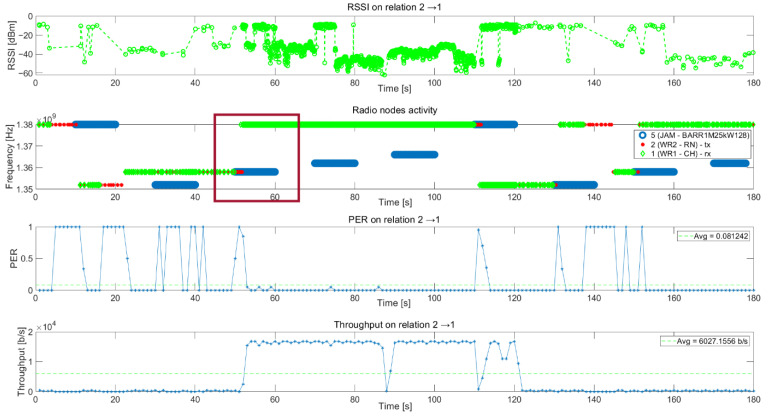
Metrics of transmission between node 2 (TX) and 1 (RX).

Figure 15 presents graphs of metrics collected from all nodes after the scenario realization. The first graph presents the RSSI of the packet received by node 1. During the tests, automatic gain control (AGC) was enabled in a fast mode in all nodes. Therefore, this level cannot be associated with radio channel attenuation.

The second graph in Figure 15 presents the packets received by node 1 from node 2 (green points), packets transmitted by node 2 to node 1 (red points), and jammer activity (blue points). On the Y-axis, available frequency channels are presented; the X-axis shows the scenario time. During valid transmission, green and red points should overlap. When the jammer started jamming, the network reacted and switched the frequency to the next data channel from the backup list (dark red rectangle).

During the scenario, five collisions can be observed between network propagation and jammer activities. In this case, there is no communication between all or some nodes. The CH has to make a decision, based on sensing information from all nodes, to stay on the network channel or to choose a better channel to recover communication in the network. During the channel change, the CH sends a packet to all nodes with a request to change a channel and all available regular nodes change their channel. If they cannot receive packets correctly from the CH node, they try to find communication on other channels, starting from the best channels from channel list.

The next two graphs show PER and throughput for this relationship. Sometimes, PER is equal to 1 (for example between 30 s and 42 s), even though there is no jamming signal on the transmission frequency. This is caused by interference from the jammer signal in the neighboring channel and the very short distance (about 15 m) between the jammer and receiver.

**Figure 16:**
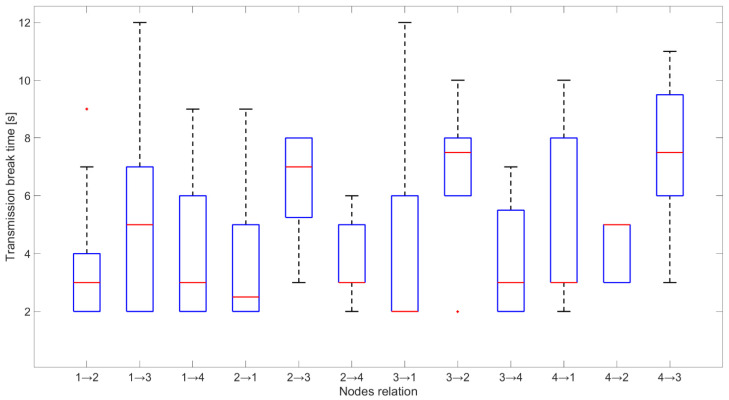
Time of node synchronization for each relationship.

Figure 16 presents the time of network synchronization on the data channel. This time is between 2 and 5 s, and it mostly equals to approximately 3 s (median value presented by the red line). These results are in line with the assumptions. Greater values are associated with signal reception problems caused by propagation channel impact or RF fronted imperfection. The number of data channel changes by the network depended mainly on the jamming activity and other interferences from radio sources not defined in the scenario.

**Figure 17:**
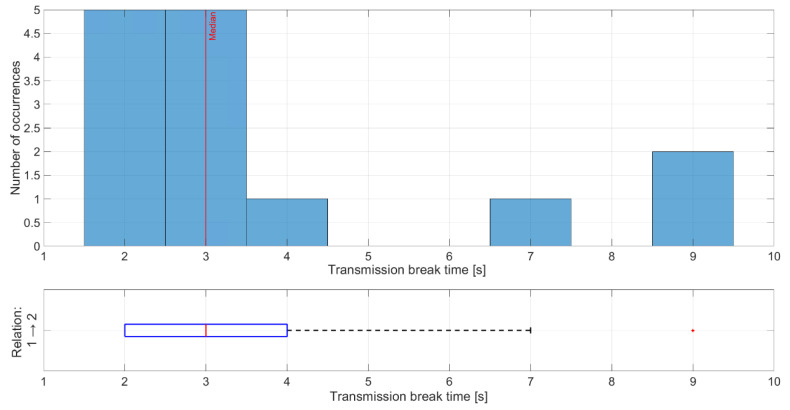
Distribution of transmission time breaks for relationship 1 and 2.

**Figure 18:**
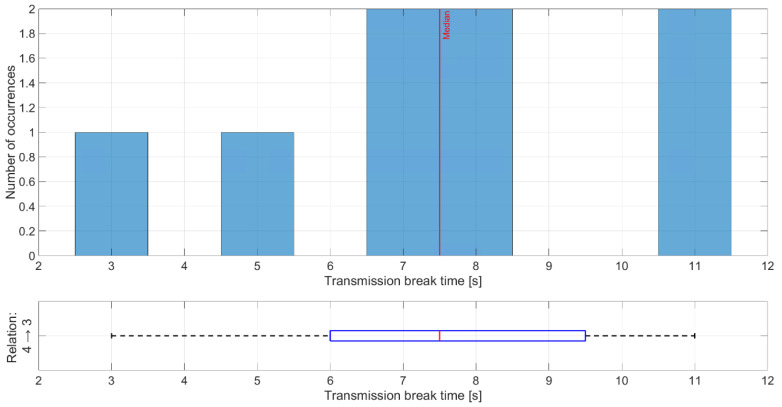
Distribution of transmission time breaks for relationship 4 and 3.

**Figure 19:**
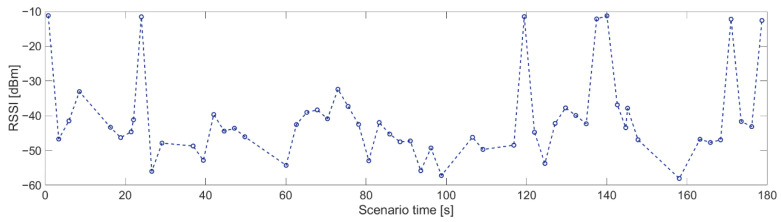
RSSI level for relationship 4 and 3.

The shortest time of transmission breaks happens between nodes 1 and 2 (Figure 17), where node 1 transmitted data to node 2. Breaks with durations of 2 and 3 s occurred five times, which is equal to the number of collisions between network transmission and the jamming signal. Longer breaks also occurred but they were caused by propagation conditions, and nodes needed longer time to recover their synchronization. Short distances between these nodes and mostly good propagation channel conditions lead to synchronization between nodes very quickly. The worst situation can be observed for the relationship between nodes 4 and 3 where the synchronization time is between 7 and 8 s (Figure 18). This is due to two reasons. First, node 4 sent only HELLO messages to node 3 every 2.5 s, so this is a minimum time when node 3 can establish a link state for this relationship. Second, the propagation conditions are unfavorable because the signal from node 4 was received at a low RSSI level (Figure 19). RSSI was calculated in the OFDM demodulator. It shows an impact of the radio channel on the received radio signal, even if AGC was enabled to improve performance. The scenario was performed in an urban area, where multipath effects were expected. Additionally, the low power of the transmitted signal resulted in power being at the receiver sensitivity limit. Some packets were not received correctly, which extended the synchronization time between individual nodes.

## 7. Conclusions

In this paper, a waveform solution elaborated for Cognitive Radio based on MANET is presented. This waveform was created using OFDM modulation and 802.15.4 MAC frames [43], modified for the requirements of cognitive solutions. The main requirement we assumed was to use the Opportunity Spectrum Access procedures to minimize the interference intra clustered network and to avoid intentional jamming. The decisions of frequency channel usage are made by Custer Head nodes based on the channel ranking list. The channel rank is calculated with regard to the fitness parameter that is a weighted function of channel distances, channel occupancy, and link quality metrics. These metrics are calculated by the learning algorithm using a history of measures based on sensing.

The major differences between the proposed solution and existing standards (Table 2) are the possibility of operation in a wide frequency range, up to 1 km range links, and immunity against strong and intentional jamming. A wide frequency range enables operation not only in ISM bands, but also in dedicated frequency channels assigned to government and military users. It allows us to achieve large spectral diversity and to avoid crowded bands and jammed channels.

An extended communication range is also necessary to conduct security and crisis-oriented operations, where access to infrastructure is not possible and distances between users are relatively large. Such extension requires both increased output power and adapted channel equalizer with the capability to cope with larger delays of reflected signals.

Additional capability is increased resistance against intentional jamming. It is achieved by cooperative spectrum sensing, providing information about the quality of unused channels. Information concerning the best channels is distributed in the network in a proactive way before interferences occur, so in the case of a lack of communication, all nodes know which channels should be used to restore the network operation and the searching procedure is much faster than in other standards. To further increase the immunity against jamming, implemented spectrum monitoring can be performed in a flexible way, covering large number of frequency channels in a wide frequency range using one radio interface. The control protocol is also redundant and resistant to transmission losses.

The achieved solution shows that the CR network with dynamic spectrum access is robust and enables us to efficiently avoid interferences. The elaborated waveform may be treated as a further extension of the existing standards.

The main contributions of the paper are:

- adaptation of 802.15.4 MAC frames for cognitive spectrum management,
- proposal of multi-channel sensing for devices with one radio frequency interface,
- elaboration of the best channel selection method for optimal spectrum access,
- creation of a testbed for MANET waveform development.

This solution was implemented using SDR USRP devices and tested in a real environment. It enabled an assessment of the influence of propagation phenomena and existing sources of interferences on the proposed algorithms. The presented results of field tests show very good behavior of our CR in a mobile environment. The proposed algorithms enable fast reaction in the presence of jamming signals, that significantly increase the network performance.

The authors’ current works are devoted to deploying the MANET CR waveform for a multi-band network. New procedures for sensing and a cognitive engine are also under investigation including a Radio Environment Map as a Database supporting cognitive manager decisions. In the future, these new functionalities will be integrated with the existing CR demonstrator to show the benefits of the DSA in real situations with intense spectrum use and for cases when intentional jamming occurs.

## Figures and Tables

**Table 1:** Cognitive waveform parameters.

Parameter	Value
Sampling rate	2 MHz
Signal bandwidth	1 MHz
Modulation	OFDM (QPSK)
TDMA frame duration	50 ms
Time slot duration	5 ms
Guard time	1 ms

**Table 2:** Comparison of the proposed solution with relevant standards.

Waveform	PHY	MAC	Frequency Range	Bandwidth	Interference Avoidance
Proposed solution	OFDM (QPSK)	TDMA	0.2–2.4 GHz	1 MHz	Cooperative sensing of used and out-band channels, packet delivery ratio analysis, proactive backup channel selection
802.15.4	O-QPSK, MPSK, BPSK, GFSK	CSMA/CA, TDMA + FHSS (TSCH)	2.4 GHz 915 MHz 868 MHz	2 MHz	Channel sensing, FHSS
802.11b	DSSS/FHSS	CSMA/CA	2.4 GHz	22 MHz	Channel sensing, FHSS
802.11g	OFDM	CSMA/CA	2.4 GHz	20 MHz	Channel sensing, adaptive modulation, and coding selection
802.11p	OFDM	CSMA/CA	5.9 GHz	5/10/20 MHz	Channel sensing
802.11ah	OFDM (BPSK, QPSK, QAM)	Restricted Access Window	sub 1 GHz bands	1, 2, 4, 8, 16 MHz	Channel sensing

## Data Availability

The data presented in this study are available on request from the corresponding author.

## Abbreviations

The following abbreviations are used in this manuscript:

AGC	Automatic Gain Control
BFT	Blue Force Tracking
BW	Basic Waveform
CH	Cluster Head
CM	Cognitive Manager
CR	Cognitive Radio
CRC	Cyclic Redundancy Check
CSMA/CA	Carrier Sense Multiple Access with Collision Avoidance
DDOS	Distributed Denial-of-Service
DSA	Dynamic Spectrum Access
DSSS	Direct Sequence Spread Spectrum
ED	Energy Detector
EOAODV	Enhancement of Opportunistic Ad-hoc On Demand Distance Vector
ETC	Expected Transmission Count
FER	Frame Error Rate
FH	Frequency Hopping
FHSS	Frequency-Hopping Spread Spectrum
GW	Gateway
HMM	Hidden Markov Model
IoT	Internet of Things
LEACH	Low Energy Adaptive Clustering Hierarchy
M2LSB	Min-Max Scheduling Load Balancing
MAC	Medium Access Control
MANET	Mobile ad hoc networks
MANET-CR	Mobile ad hoc networks with Cognitive Radio
OFDM	Orthogonal Frequency Division Multiplexing
PER	Packet Error Rate
PHY	Physical layer
QoS	Quality of Service
RN	Regular Node
RSSI	Received Signal Strength Indicator
SDR	Software Defined Radio
SEP	Stable Election Protocol
SM	Sensing Module
SRF	Sensing Results Fields
TDMA	Time Division Multiple Access
TSCH	Time Slotted Channel Hopping
UHD	USRP Hardware Driver
USRP	Universal Software Radio Peripheral
WCNE	Weighted Clusterhead Node Election algorithm
WOLA	Weighted Overlap-Add
WSN	Wireless Sensors Networks
